# Expansion of viral variants associated with immune escape and impaired virion secretion in patients with HBV reactivation after resolved infection

**DOI:** 10.1038/s41598-018-36093-w

**Published:** 2018-12-24

**Authors:** Tadashi Inuzuka, Yoshihide Ueda, Soichi Arasawa, Haruhiko Takeda, Tomonori Matsumoto, Yukio Osaki, Shinji Uemoto, Hiroshi Seno, Hiroyuki Marusawa

**Affiliations:** 1 Department of Gastroenterology and Hepatology, Graduate School of Medicine, Kyoto University, Kyoto, Japan; 2 Research Center for Hepatitis and Immunology National Center for Global Health and Medicine, Chiba, Japan; 3 Research Fellow of Japan Society for the Promotion of Science, Tokyo, Japan; 4 Department of Gastroenterology and Hepatology, Osaka Red Cross Hospital, Osaka, Japan; 5 Department of Surgery, Graduate School of Medicine, Kyoto University, Kyoto, Japan

## Abstract

HBV reactivation could be induced under immunosuppressive conditions in patients with resolved infection. This study aimed to clarify the viral factors associated with the pathogenesis of HBV reactivation in association with the immunosuppressive status. Whole HBV genome sequences were determined from the sera of 24 patients with HBV reactivation, including 8 cases under strong immunosuppression mediated by hematopoietic stem cell transplantation (HSCT) and 16 cases without HSCT. Ultra-deep sequencing revealed that the prevalence of genotype B and the ratio of non-synonymous to synonymous evolutionary changes in the surface (S) gene were significantly higher in non-HSCT cases than in patients with HSCT. Those non-synonymous variants included immune escape (6/16 cases) and MHC class II-restricted T-cell epitope variants (6/16 cases). Furthermore, reactivated HBV in 11 of 16 (69%) non-HSCT cases possessed substitutions associated with impaired virion secretion, including E2G, L77R, L98V, T118K, and Q129H in the S region, and M1I/V in the PreS2 region. In conclusion, virologic features of reactivated HBV clones differed depending on the intensity of the immunosuppressive condition. HBV reactivation triggered by immunosuppressive conditions, especially those without HSCT, was characterized by the expansion of variants associated with immune escape, MHC class II-restricted T-cell epitope alterations, and/or impaired virion secretion.

## Introduction

Hepatitis B virus (HBV) infection is a major global health problem, and approximately 257 million people are estimated to be carriers of hepatitis B surface antigen (HBsAg)-positive HBV^1^. HBV persists in the liver after the disappearance of HBsAg in individuals with previous exposure to the virus, maintaining the serologic footprint of the antibody to hepatitis B core antigen (anti-HBc) positivity^2^. An estimated 2 billion people worldwide with resolved HBV infection are serologically HBsAg-negative and anti-HBc–positive^3^. Accumulating evidence indicates that individuals with resolved infection carry a risk for HBV reactivation when they receive immunosuppressive therapy or cytotoxic chemotherapy^4^. Therefore, screening for not only HBsAg but also anti-HBc and/or the antibody to HBsAg (anti-HBs) is essential before administering immunosuppressive therapy or cytotoxic chemotherapy, and monitoring HBV DNA and early intervention with nucleos(t)ide analogues reduces hepatitis caused by HBV reactivation in individuals with resolved infection^5,6^.

The frequencies of HBV reactivation during/after immunosuppressive therapy or cytotoxic chemotherapy vary according to the host immune system and HBV infection status. Previous studies reported the clinical factors associated with HBV reactivation in patients with resolved infection, which include male sex, disease type, and those that were low anti-HBs and/or high anti-HBc before viral reactivation^7–9^. The intensity of immunosuppression is also likely to have a decisive influence on the risk of viral reactivation. Indeed, patients treated with hematopoietic stem cell transplantation (HSCT) carry the highest risk for HBV reactivation, with frequencies ranging from 14% to 41%^6,10^. Patients treated with HSCT have the highest risk of HBV reactivation probably due to strong immunosuppression, but also to the loss of their previously acquired immunity against HBV^11^. Little is known, however, about the association between the types of immunosuppressive therapy and the virologic factors in the pathogenesis of HBV reactivation in patients with resolved infection.

We previously demonstrated that resolved HBV carriers with the G1896A variant (guanine to adenine mutant at nucleotide 1896) in the precore gene of the HBV genome might have an increased risk of HBV reactivation and fatal acute liver failure^12^. Our findings suggested that not only host factors, but also viral factors, play a critical role in the risk of HBV reactivation from resolved infection under immunosuppressive conditions. Consistently, recent studies demonstrated that immune escape variants with specific amino acid substitutions in the major hydrophilic region in the small surface (S) protein encoded by the S gene of the HBV genome were detectable in the sera of patients with HBV reactivation^7,13^. In addition, substitutions with an additional N-linked glycosylation (NLG) site, which cause the addition of glycans to asparagine residues, were detectable in the small S protein in a subset of patients with viral reactivation^13^. Whether variants with altered S protein emerge after viral exacerbation or whether individuals with resolved HBV infection having those variants are at high risk for viral reactivation, however, is not clear.

In the present study, we used an ultra-deep sequencing technique to clarify the significance of viral variants in the pathophysiology of HBV reactivation in patients with resolved infection. To determine the virologic factors associated with various types of immunosuppressive therapy, we compared the viral genome in patients with resolved HBV infection who received immunosuppressive therapy or cytotoxic chemotherapy with and without HSCT. We also examined the characteristics of the viral genome in the liver of individuals with resolved HBV infection who did not experience viral reactivation.

## Results

### Reactivation of genotype B HBV was prevalent in patients under immunosuppressive conditions without HSCT

This study included 24 patients with a diagnosis of HBV reactivation after resolved infection, including 16 patients (67%) who underwent immunosuppressive therapy or cytotoxic chemotherapy without HSCT (non-HSCT group) and 8 patients (33%) who underwent HSCT (HSCT group) prior to HBV reactivation (Table 1). In total, 17 of 24 (71%) patients were male, with a median age of 66 (range, 25–87) years. The median patient age was significantly younger in the HSCT group than in the non-HSCT group (59 vs 73, respectively; *p* < 0.01). Of the 24 patients, 21 (88%) had a hematologic malignancy, including 11 patients with malignant lymphoma, 5 with multiple myeloma, 4 with acute leukemia, and 1 with primary macroglobulinemia. The remaining three patients had colon cancer, lung cancer, and psoriasis, respectively. Before HBV reactivation, all patients were originally HBsAg-negative, but anti-HBc-positive. Of the 24 patients, 8 (33%) were anti-HBs-positive, 7 (29%) were anti-HBs-negative, and in 9 (38%) patients, the anti-HBs status before viral reactivation was unknown. Pre-reactivation sera from 12 patients were available for further analysis, and confirmed that serum HBV DNA was undetectable in the repeated PCR. Other than the median age, the clinical factors did not differ significantly between the non-HSCT and HSCT groups.

**Table 1:** Clinical characteristics of the patients with HBV reactivation and acute hepatitis B.

Patients’ Characteristics	non-HSCT group	HSCT group	AHB group
	(n = 16)	(n = 8)	(n = 23)
Gender (M/F)	11/5	6/2	15/8
Median age (years)	73 (52–87)	59 (25–66)	41 (21–76)
Primary disease			
Malignant lymphoma	9 (56%)	2 (25%)	—
Multiple myeloma	2 (13%)	3 (38%)	—
Leukemia	1 (6%)	3 (38%)	—
Chronic inflammatory diseases	1 (6%)	0 (0%)	—
Other diseases	3 (19%)	0 (0%)	—
Immunosuppressive therapy, N (%)			
Hematopoietic Stem Cell Transplantation	0 (0%)	8 (100%)	—
Rituximab-containing chemotherapy	10 (63%)	1 (13%)	—
Other cytotoxic chemotherapy	5 (31%)	0 (0%)	—
Other immunosuppressive therapy	1 (6%)	0 (0%)	—
Prereactivation HBV status, N (%)			
Isolated anti-HBc positive	5 (31%)	2 (25%)	—
Anti-HBc positive/anti-HBs positive	3 (19%)	5 (63%)	—
Anti-HBc positive/unknown anti-HBs status	8 (50%)	1 (13%)	—
Median HBV DNA, log IU/ml	5.6 (1.7–8.2)	7.5 (2.7–8.2)	4.5 (1.8–8.2)
Median ALT, IU/l	68 (10–2028)	29 (15–1915)	1992 (404–4062)
Median white blood cell counts			
White blood cell counts, /µl	4140 (1100–22620)	5750 (4050–7100)	5980 (3790–15870)
Neutrophil counts, /µl	2540 (240–20810)	3440 (2800–5400)	3300 (1800–8760)
Lymphocyte counts, /µl	1050 (0–3510)	1260 (320–1900)	1930 (1050–5520)
HBV subgenotype			
B1/Bj	7 (44%)	0 (0%)	1 (4%)
B2/Ba	2 (13%)	0 (0%)	2 (9%)
C1/Cs	0 (0%)	0 (0%)	2 (9%)
C2/Ce	7 (44%)	8 (100%)	18 (78%)

At the time of diagnosis with HBV reactivation, the median HBV-DNA level was 5.6 (range, 1.7–8.2) log IU/ml in the non-HSCT group and 7.5 (range, 2.7–8.2) log IU/ml in the HSCT group. The median alanine transaminase (ALT) level was 68 (range, 10–2028) IU/l in the non-HSCT group and 29 (range, 15–1915) IU/l in the HSCT group. The median white blood cell counts, neutrophil counts and lymphocyte counts were 4140/µl (range, 1100–22620/µl), 2540/µl (range, 240–20810/µl) and 1050/µl (range, 0–3510/µl), respectively, in the non-HSCT group and 5750/µl (range, 4050–7100/µl), 3440/µl (range, 2800–5400/µl) and 1260/µl (range, 320–1900/µl), respectively, in the HSCT group. In this study, we investigated the viral genome of 23 patients with acute hepatitis B (AHB) as a control. At the time of diagnosis with AHB, the median HBV-DNA level was 4.5 log IU/ml (range, 1.8–8.2 log IU/ml) and the median ALT level was 1992 IU/l (range, 404–4062 IU/l). The median white blood cell counts, neutrophil counts and lymphocyte counts were 5980/µl (range, 3790–15870/µl), 3300/µl (range, 1800–8760/µl) and 1930/µl (range, 1050–5520/µl), respectively, in the AHB group. The HBV-DNA level in the AHB group at the time of diagnosis was significantly lower than that in the non-HSCT and HSCT groups (*p* < 0.01). On the other hand, the ALT level in the AHB group was significantly higher than that in the non-HSCT and HSCT groups (*p* < 0.01). The neutrophil count was not significantly different between any two groups among the non-HSCT, HSCT, and AHB groups. The white blood cell count in the AHB group was significantly higher than that in the non-HSCT group (*p* < 0.05), and the lymphocyte count in the AHB group was significantly higher than that in the non-HSCT and HSCT groups (*p* < 0.05). We first determined the genotype/subgenotype of the reactivated viruses based on the HBV genome sequence in each case. Of note, the phylogenetic analyses revealed that 9 of 16 (56%) cases in the non-HSCT group had reactivation of genotype B viruses, including 7 subgenotype B1/Bj and 2 subgenotype B2/Ba (Table 1 and Supplementary Fig. S3). In contrast, none of the cases in the HSCT group had genotype B, but all had genotype C (subgenotype C2/Ce). The circulating HBV in the AHB group showed genotype B infection in only 3 of 23 (13%; 1 was subgenotype B1/Bj and 2 were subgenotype B2/Ba), and the remaining 20 (87%) were genotype C HBV (2 were subgenotype C1/Cs and 18 were subgenotype C2/Ce). These findings indicated that reactivation of genotype B HBV was prevalent in patients with resolved infection that received immunosuppressive therapy and/or cytotoxic chemotherapy without HSCT.

### Non-synonymous variants were accumulated in the S gene of the reactivated HBV especially in patients without HSCT

Ultra-deep sequencing determined viral genome sequences with a mean 53,636-fold coverage at each nucleotide site for serum specimens from non-HSCT cases, a mean 62,132-fold coverage for those from HSCT cases, and a mean 48,025-fold coverage for those from AHB cases (Supplementary Table S1). As we reported previously, the genetic heterogeneity of the viruses extracted from patients with HBV reactivation was quite low in each case^12^. In fact, the median number of nucleotide variants that accounted for more than 5% and 20% of the heterogeneity was 7.5 bases (0.2%) [range, 0–79 bases (0–2.5%)] and 2.5 bases (0.08%) [range, 0–44 bases (0–1.4%)], respectively, in 3215 whole bases (Supplementary Table S2). From this result, we selected the major strain as the representative sequence in each case by ultra-deep sequencing and used it for the following analyses.

**Figure 1:**
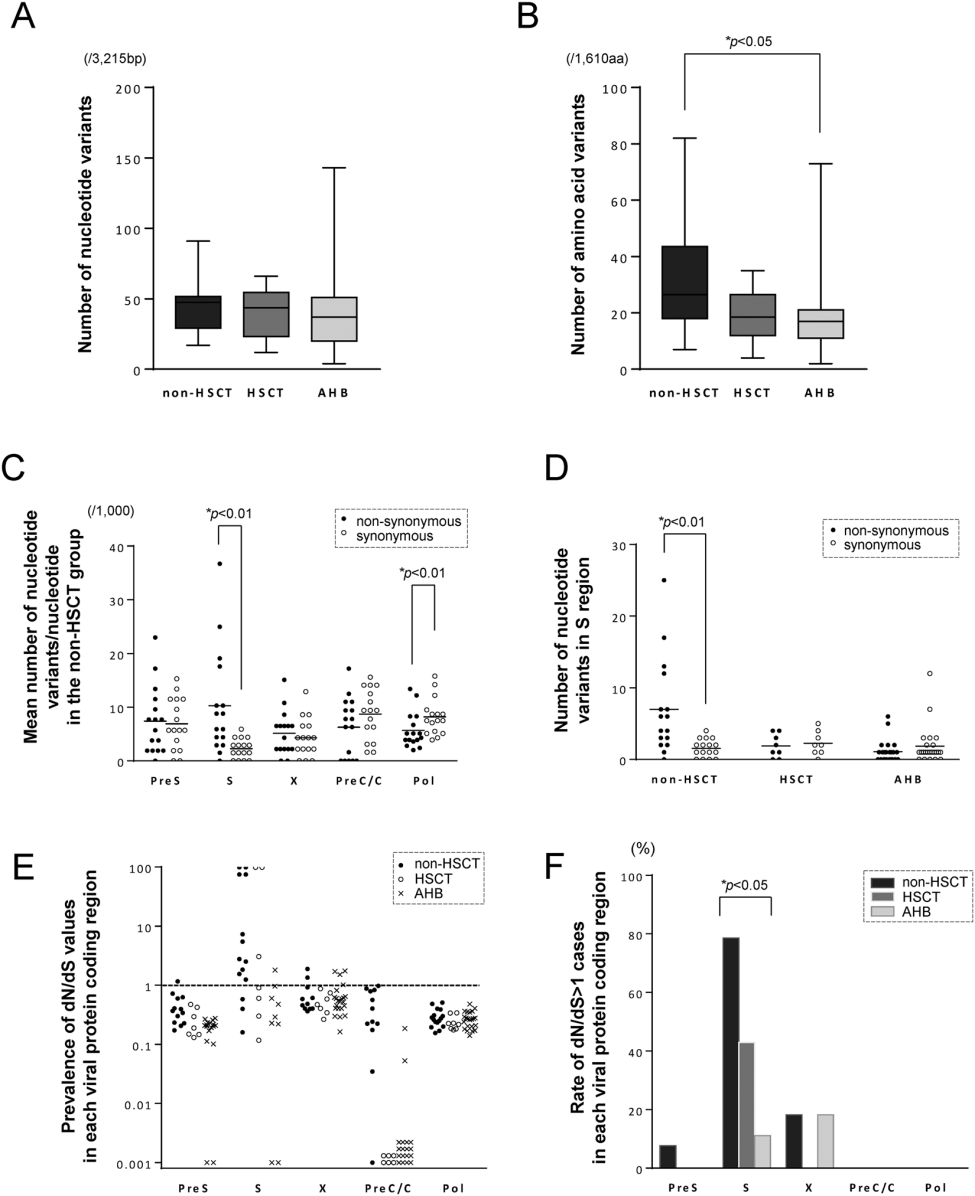
Comparison of nucleotide and amino acid variants among patients with HBV exacerbation in the non-HSCT, HSCT, and AHB groups. Difference in the number of nucleotide variants in the full HBV genome (**A**) and amino acid variants (**B**) among the non-HSCT, HSCT, and AHB groups (Kruskal-Wallis test and following Steel-Dwass test for post hoc analysis). The PreS represents the domain that is coded by the PreS1 and PreS2 genes. (**C**) The mean number of nucleotide variants per nucleotide in each HBV protein coding region of viral genome in the non-HSCT group and (**D**) the total number of nucleotide variants in the S gene among the three groups. The bar represents the median value. The asterisk indicates a statistically significant difference between the synonymous and non-synonymous variants (*p* < 0.01, Mann–Whitney *U* test). (**E**) The dN/dS values of each viral protein among the three groups. (**F**) The frequencies of cases whose dN/dS ratio was greater than one in each viral protein among the three groups. The asterisk indicates a statistically significant difference among the three groups (*p* < 0.05, chi-square test). Abbreviations: AHB, acute hepatitis B, HSCT, hematopoietic stem cell transplantation, Pol, polymerase, PreC/C, precore/core protein, PreS, presurface, S, surface, X, X protein.

First, we confirmed that there was no significant difference in the total number of nucleotide variants of the HBV genome among the non-HSCT, HSCT, and AHB groups (Fig. 1A). We then examined the proportion of non-synonymous variants among the total nucleotide variants in the reactivated viruses of each group. Because HBV partially overlaps four open reading frames encoding the surface antigen, nucleocapsid, X protein (X), and polymerase (Pol), we separately estimated the total number of amino acid variants in the presurface (PreS) domain, S protein, X, precore/core (PreC/C), and Pol coding regions of the HBV genome. As shown in Fig. 1B, the total number of amino acid variants was significantly greater in the reactivated viruses of the non-HSCT group than in those of the AHB group (*p* < 0.05). In addition, amino acid variants tended to be more abundant in the non-HSCT group than in the HSCT group, although the difference was not statistically significant (*p* = 0.25).

To clarify the viral proteins that contained amino acid variants in the reactivated viruses, we determined the distribution of non-synonymous and synonymous variants in each HBV protein coding sequence in the non-HSCT cases. Synonymous variants were predominant in the Pol regions (*p* < 0.01), and there was no significant difference between the non-synonymous and synonymous variants in the PreS, X, and PreC/C regions. In contrast, non-synonymous variants were prevalent in the S region of the reactivated viruses (*p* < 0.01; Fig. 1C). To compare the frequencies of amino acid variants in the small S protein among the non-HSCT, HSCT, and AHB groups, we examined the number of non-synonymous and synonymous variants in the S region of the viruses in each group. Non-synonymous variants in the S region were prevalent in the viruses from the non-HSCT group compared with the HSCT and AHB groups (Fig. 1D). To estimate the putative selective pressure in the reactivated HBV of non-HSCT cases, we determined the non-synonymous (dN) and synonymous (dS) substitution rates (dN/dS ratios) in each HBV protein-coding region. The dN/dS values of viruses can be greater than one when they are subject to positive selection (adaptive evolution)^14^. Of note, viruses with a dN/dS ratio greater than one were prevalent in the S region among all HBV proteins, especially in non-HSCT cases (Fig. 1E). Namely, in the non-HSCT group, 11 of 14 (79%) cases had HBV S regions with a dN/dS ratio greater than one (Fig. 1F). In contrast, in the HSCT and AHB groups, three of seven (43%) and one of nine (11%) cases, respectively, had circulating viruses with a dN/dS ratio greater than one in the S region. These findings suggest that viruses with non-synonymous variants in the S region were positively selected, especially in patients with HBV reactivation in the non-HSCT group.

### High prevalence of HBV with immune escape and/or MHC class II-restricted T-cell epitope variants in the reactivated viruses

**Figure 2:**
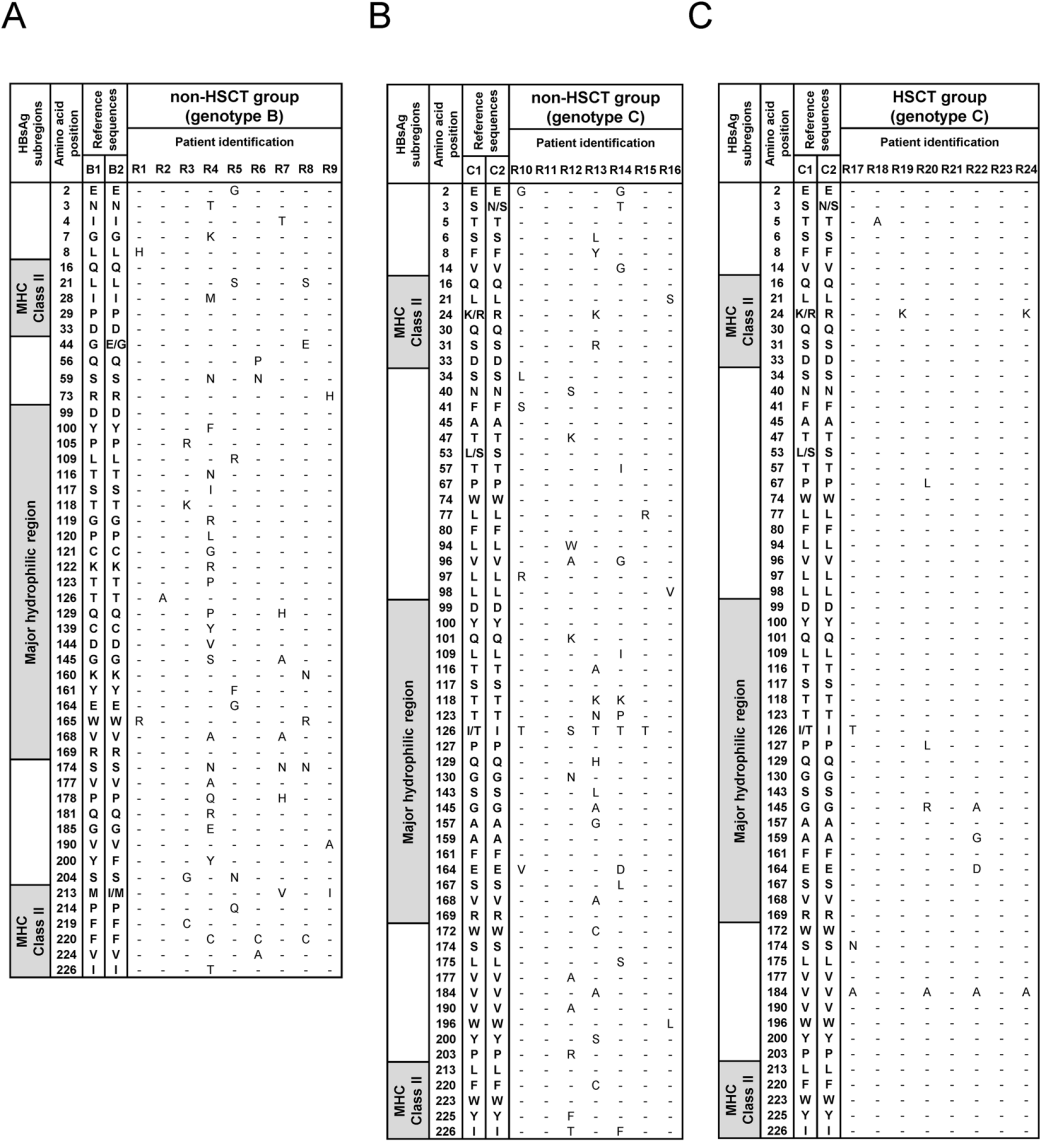
Prevalence of HBV variants in the small S protein in patients with HBV reactivation under immunosuppressive therapies and/or cytotoxic chemotherapies. Amino acids harbored by each reference sequence are shown on the left side. Only positions with amino acid substitutions as compared to HBV reference sequences are shown, and the dash indicates the absence of an amino acid substitution. R1-24 are the cases with HBV reactivation. MHC class II on the left side means the MHC class II-restricted T-cell epitope region. (**A**) Patients with genotype B HBV in the non-HSCT group. (**B**) Patients with genotype C HBV in the non-HSCT group. (**C**) Patients with genotype C HBV in the HSCT group. Abbreviation: HSCT, hematopoietic stem cell transplantation.

All amino acid variants detected in the small S protein of 24 patients at HBV reactivation are shown in Fig. 2. First, we detected a number of amino acid substitutions in the major hydrophilic region, including the amino acid 124–147 region encoding the dominant neutralizing B-cell epitope defined as the ‘a’ determinant region in non-HSCT cases^15^. Amino acid substitutions associated with immune escape, such as sT118K (n = 3), sG145A (n = 3), and sG145R (n = 1) variants^13,15–20^, were abundant, especially in non-HSCT patients at HBV reactivation. Moreover, three patients in the non-HSCT group had predominant variants with sT116N, sT123N, and sG130N changes, which resulted in the addition of NLG sites by substitutions of threonine or glycine with asparagine. These additional NLG sites in the small S protein associate with impaired HBsAg recognition of anti-HBs antibodies caused by the masking B-cell epitope^13,19,21^.

Next, we also identified amino acid substitutions sL21S (n = 3) and sF220C (n = 4) in the MHC class II-restricted T-cell epitope region, which are reported to cause a complete loss of T-cell reactivity and anti-HBs production among individuals vaccinated against HBV in an *ex vivo* study^22^. All six patients with MHC class II-restricted T-cell epitope substitutions were in the non-HSCT group, while none of the patients in the HSCT and AHB groups had MHC class II-restricted T-cell epitope substitutions (Figs 2 and S1).

**Figure 3:**
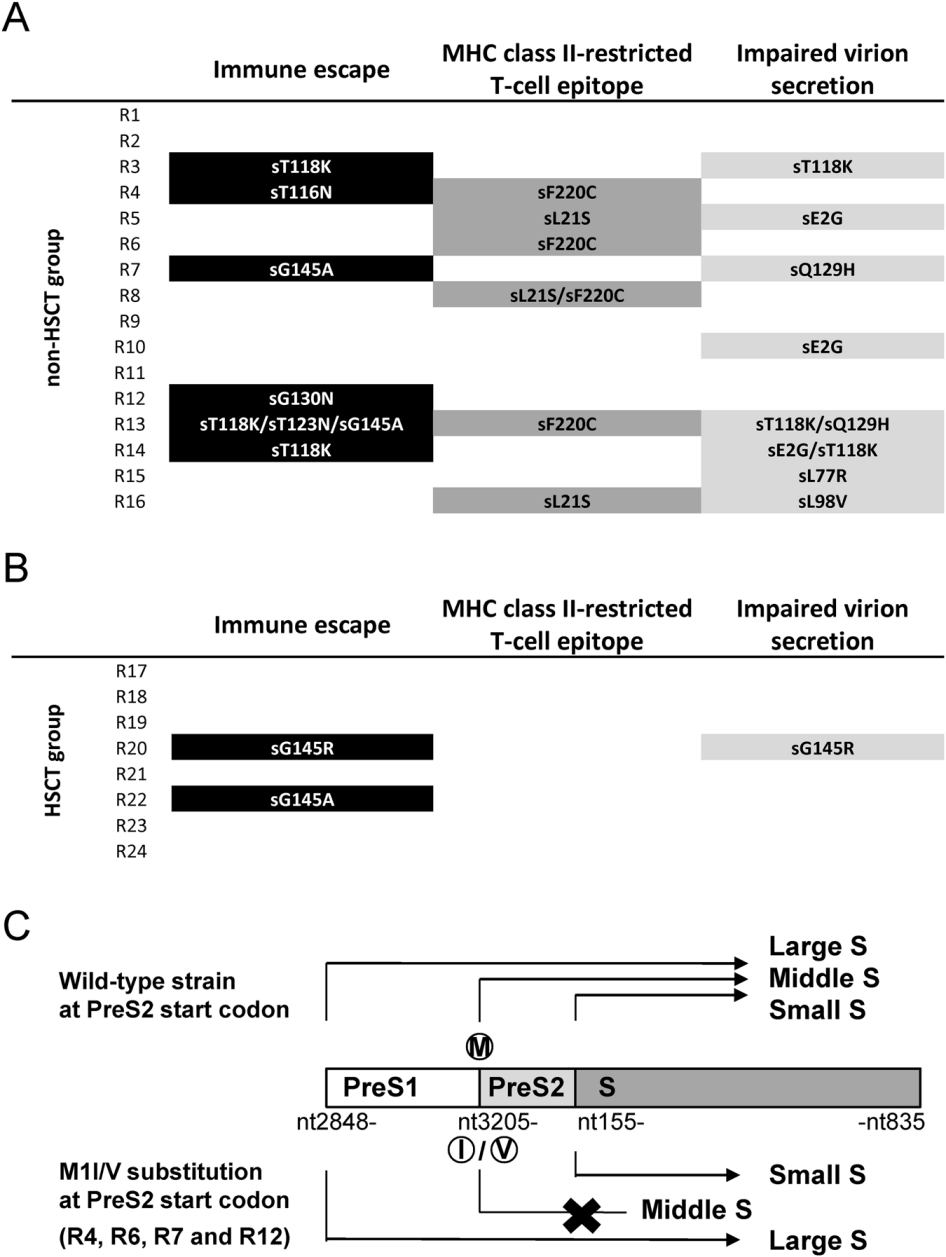
HBV variants with immune escape, MHC class II-restricted T-cell epitope alterations and/or impaired virion secretion in patients with HBV reactivation. The prevalence of characteristic amino acid substitutions in the small S protein (**A**) in the non-HSCT and (**B**) HSCT groups. R1-24 are the cases with HBV reactivation. (**C**) The reactivated viral clones in four cases (R4, R6, R7, and R12) of the non-HSCT group were variants with the M1I/V substitution at the PreS2 start codon, which resulted in the ablation of the middle S protein. Abbreviation: HSCT, hematopoietic stem cell transplantation.

In total, 10 of 16 (63%) cases in the non-HSCT group had a dominant population of variants associated with immune escape and/or MHC class II-restricted T-cell epitope alterations, which could affect the adaptive immune system (Fig. 3A). In contrast, in the HSCT group, only two of eight (25%) cases had immune escape variants and none of those cases possessed MHC class II-restricted T-cell epitope variants (Fig. 3B). No patients in the AHB group had any variants in those regions (Supplementary Fig. S1). These findings suggest that variants with putative escape from the immune response against HBV play a critical role in viral reactivation, especially in patients that receive immunosuppressive therapy or cytotoxic chemotherapy without HSCT.

### Variants with a phenotype of impaired virion secretion were prevalent in patients with HBV reactivation

Previous studies demonstrated that several variants in the S region of the HBV genome could lead to reduced secretion of virions from the infected hepatocytes^17,23^. In addition, several variants in the immunodominant loop of surface protein, including sT118K, sQ129H, and sG145R substitutions in the small S protein are reported to induce impaired virion secretion from infected liver cells^17,20^. Interestingly, sT118K (n = 3) and sQ129H (n = 2) variants were predominant in patients in the non-HSCT group, and sG145R was detected in one patient in the HSCT group (Fig. 2). Amino acid substitutions of sE2G (n = 3), sL77R (n = 1), and sL98V (n = 1) in the small S protein that were associated with impaired virion secretion were also the predominant clone in patients in the non-HSCT group^24,25^. In total, the small S protein variants associated with impaired virion secretion were detected as the predominant clones in 8 of 16 (50%) patients with HBV reactivation under immunosuppression without HSCT, but in only 1 of 8 (13%) patients with HSCT (Fig. 3A,B).

**Figure 4:**
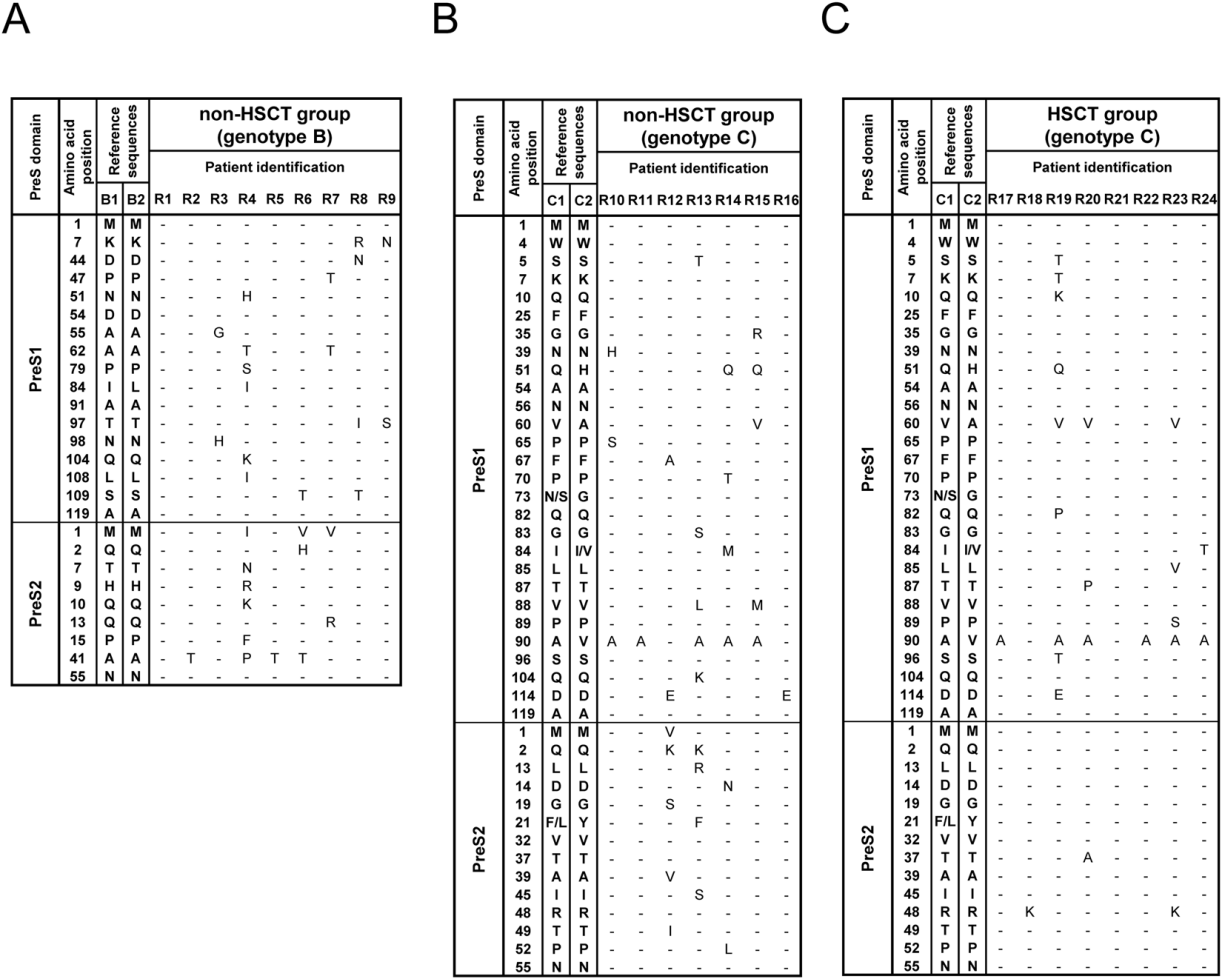
Amino acid variants of the PreS1/PreS2 coding domain in patients with HBV reactivation under immunosuppressive therapies and/or cytotoxic chemotherapies. Amino acids harbored by each reference sequence are shown on the left side. Only positions with amino acid substitutions as compared with HBV reference sequences are shown, and the dash indicates the absence of an amino acid substitution. R1-24 are the cases with HBV reactivation. (**A**) Patients with genotype B HBV in the non-HSCT group. (**B**) Patients with genotype C HBV in the non-HSCT group. (**C**) Patients with genotype C HBV in the HSCT group. Abbreviation: HSCT, hematopoietic stem cell transplantation, PreS, presurface.

The middle S protein of HBV is reported to be dispensable for virion formation and secretion^26^, and deficiency of middle S protein production could result in impaired virion secretion^27^. Interestingly, M1I/V variants at the start codon of the PreS2 gene, resulting in defective expression of the middle S protein, were also detected in the reactivated viruses of patients in the non-HSCT group. Indeed, ultra-deep sequencing analysis showed that almost all the reactivated viral clones in four cases in the non-HSCT group were variants with the M1I/V substitution at the PreS2 start codon (frequency of 99.7% to 99.9% for total viral population), suggesting that these viral clones also impaired virion secretion caused by a deficiency of middle S protein production (Figs 3C and 4). In contrast, none of the patients in the HSCT and AHB groups had those PreS2 variants in their serum at the time of HBV exacerbation (Figs 4 and S2).

Taken together, 11 of 16 (69%) patients in the non-HSCT group had a dominant population of the HBV S or PreS2 variants associated with a putative phenotype of impaired virion secretion, while only 1 of 8 (13%) patients in the HSCT group and none in the AHB group possessed the variants associated with impaired virion secretion.

### Low prevalence of viral clones with altered S protein in the liver of individuals with resolved HBV infection

**Figure 5:**
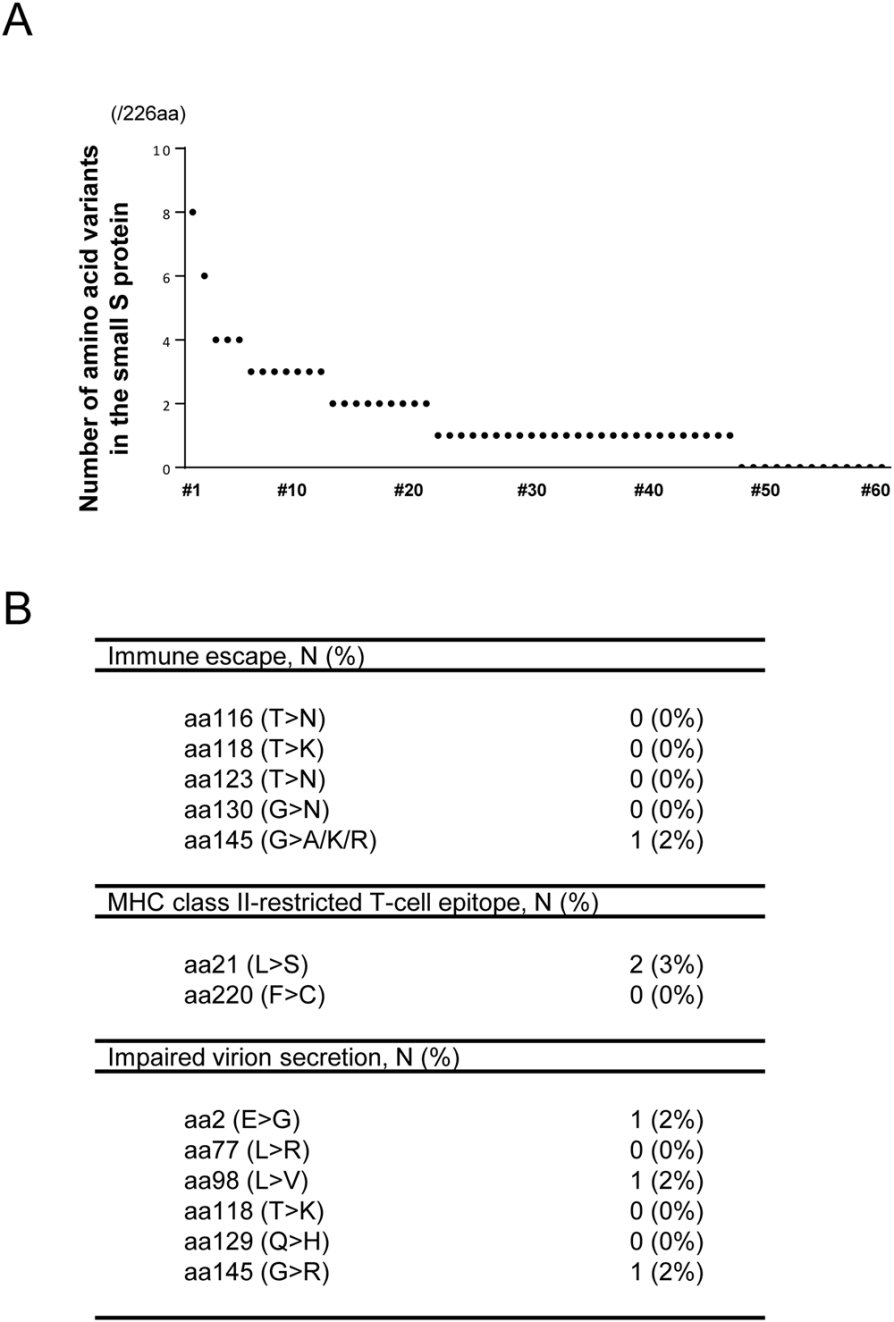
Prevalence of viral variants with altered S protein in the liver of individuals with resolved HBV infection. (**A**) The prevalence of amino acid variants in the small S protein (226 amino acids) of genotype B/C HBV infected liver in individuals with resolved infection (n = 60). (**B**) Of 60 individuals, 5 (8%) showed intrahepatic infection by variants potentially associated with either immune escape (n = 1), MHC class II-restricted T-cell epitope changes (n = 2), or reduced secretion of virions (n = 3) in their liver tissues.

To clarify whether viral clones with various variants in the S region pre-existed in the liver before reactivation or were selected for and became dominant with the acquisition of those variants after reactivation, we determined the prevalence of HBV variants in the S gene in the liver of individuals with resolved infection without viral reactivation. For this purpose, we determined the sequences of the S region of the HBV genome in the liver tissues of HBsAg-negative, but anti-HBc-positive, healthy donors for living-donor liver transplantation (LDLT). Whole S gene sequences of HBV were successfully amplified from the liver from 62 of 90 (69%) individuals with resolved infection. Ultra-deep sequencing achieved a mean coverage depth of 36,047 bp for each nucleotide site of the HBV sequences (Supplementary Table S3). The phylogenetic analyses of the HBV genome sequences revealed that 2 (3%) patients had genotype A, 3 (5%) had subgenotype B1/Bj, 5 (8%) had subgenotype B2/Ba, and the remaining 52 (84%) had subgenotype C2/Ce, indicating that the prevalence of genotype B was relatively lower than the prevalence of reactivated viral isolates in patients under immunosuppressive conditions without HSCT. To compare the prevalence of amino acid substitutions with that of the reactivated viral clones, we focused on 60 samples infected with genotype B or C HBV from the liver of individuals with resolved infection. Baseline clinical characteristics of 60 individuals with resolved infection are summarized in Supplementary Table S4. We found very few amino acid variants (median 1, range 0–8 per 226 amino acids) of the small S protein in the liver of individuals with resolved HBV infection, compared with those of the reactivated viruses in the sera of patients in the non-HSCT group (median 5, range 0–25 per 226 amino acids; Figs 2 and 5A). Further, 5 of 60 (8%) individuals had intrahepatic infection by HBV with variants potentially associated with either immune escape (n = 1), MHC class II-restricted T-cell epitope changes (n = 2), or reduced secretion of virions (n = 3) in their liver tissues with a variety of frequencies for total viral clones (median 67.3%, range, 1.9% to 99.9%; Fig. 5B and Supplementary Table S5). These findings indicate that the prevalence of HBV variants with altered small S protein in the latently infected liver was much lower than that of the reactivated viral clones under immunosuppression.

### Expansion of viral clones with mutations in the S region in an individual with resolved HBV infection

**Figure 6:**
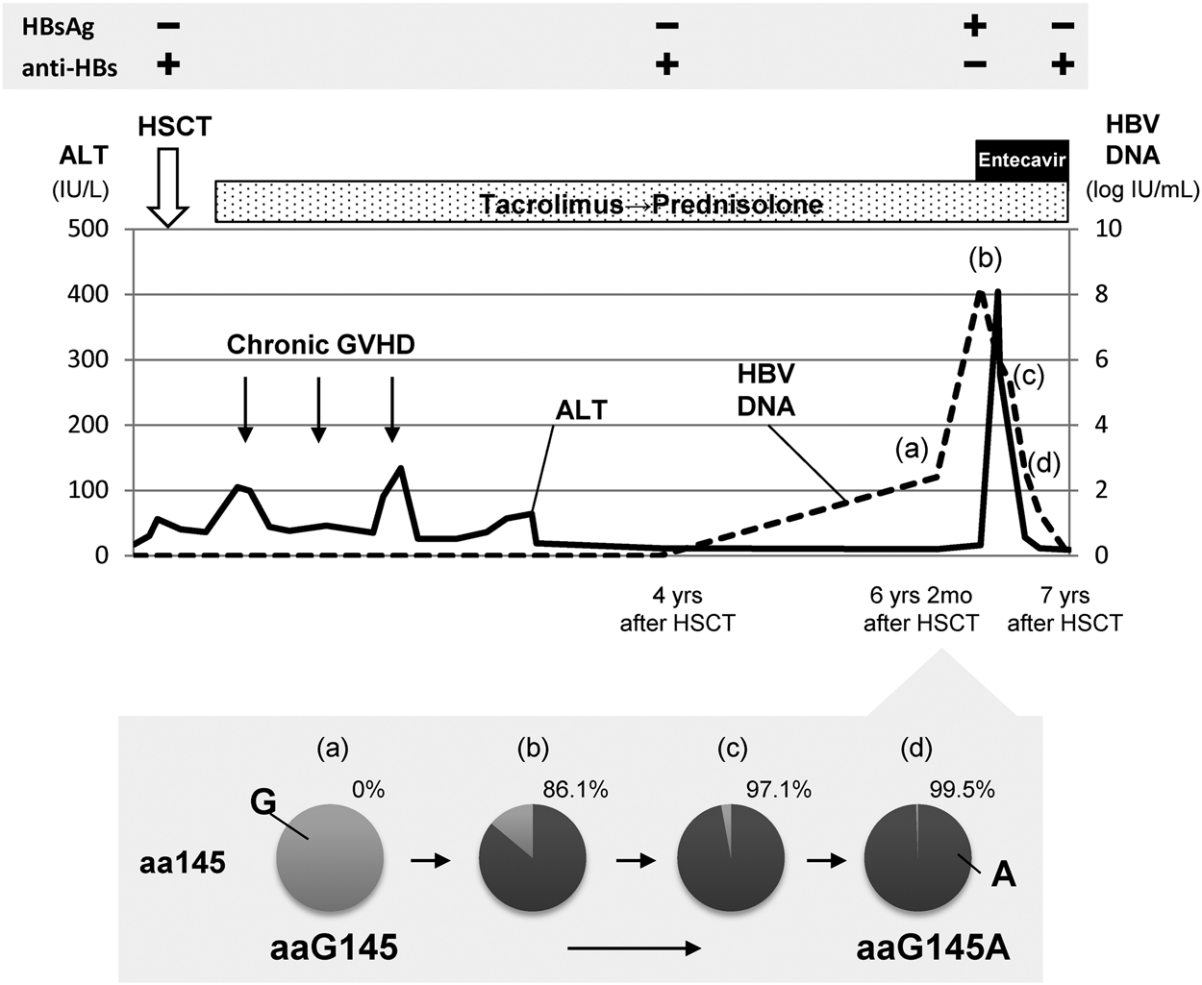
Viral dynamics of variants with altered S protein in the representative case with HBV reactivation. A case (R22) that acquired an immune escape mutation, sG145A, in the small S protein during the process of HBV reactivation. The frequency of sG145A variants in four serum samples at different time-points, analyzed by ultra-deep sequencing, is shown in the lower panel. Abbreviation: ALT, alanine transaminase, anti-HBs, antibody to HBsAg, GVHD, graft versus host disease, HSCT, hematopoietic stem cell transplantation.

We evaluated the viral dynamics of variants with altered S protein in detail in one patient whose serum samples during the time-course of HBV reactivation was available for sequencing analyses. The clinical course of this patient is shown in Fig. 6. Case R22 was originally HBsAg-negative, but anti-HBc positive, and received HSCT for a diagnosis of myelodysplastic syndrome-overt leukemia. HBV DNA in the serum became positive over 6 years after HSCT, followed by positivity for HBsAg. Among the whole HBV genome, only 3 (0.1%) of 3215 nucleotides substantially changed in the predominant viral clones during viral reactivation. These three altered nucleotides included non-synonymous mutations in the S gene coding region; sG145A, sE164D, and s*223 W (conversion of a stop codon into a tryptophan codon) alterations. Of note, the frequency of sG145A variants, representing immune escape variants, increased from 0% to 86.1% for the total viral population in accordance with an increase in HBV DNA levels from 2.4 log IU/ml to 8.2 log IU/ml. Finally, more than 99% of all the circulating HBV isolates became sG145A variants, followed by a subsequent decrease in the viral load mediated by administration of the nucleoside analogue entecavir. Variants with the sE164D substitution also increased from 0% to 99.5% for the total population, and variants with the s*223 W substitution expanded from 8.3% to 99.4% of the circulating viral isolates, while the virologic significance of these two substitutions remained unknown. Together with the low prevalence of HBV variants with altered S protein in the liver of individuals without immunosuppressive conditions, these findings suggest that several mutations associated with the anti-viral immune response might newly emerge on infected viral clones after viral reactivation under immunosuppressive conditions.

## Discussion

It is well recognized that individuals with resolved HBV infection can develop viral reactivation, so-called *de novo* HBV reactivation, in response to immunosuppressive therapy and/or cytotoxic chemotherapy. The viral factors attributed to HBV reactivation after the resolved infection are not fully understood because of the low incidence of HBV reactivation among HBsAg-negative, but anti-HBc-positive, individuals under immunosuppressive conditions. In the present study, we investigated the virologic features of reactivated HBV in patients with resolved infection in association with the type of the immunosuppressive condition.

Increasing evidence suggests that the incidence of viral reactivation differs depending on the type of the immunosuppressive condition. Immunosuppression in patients receiving HSCT is associated with an especially high risk of HBV reactivation, ranging from 14% to 41%^6,10^. In contrast, the incidence of viral reactivation in patients receiving immunosuppressive therapy and/or cytotoxic chemotherapy without HSCT was relatively low compared to that in patients with HSCT^3^. In this study, we revealed that the genotype of the reactivated HBV clearly differed between immunosuppressed patients with and without HSCT. In the non-HSCT group, 9 of 16 (56%) patients had genotype B HBV, including 7 cases with subgenotype B1/Bj viral clones. In sharp contrast to those without HSCT, no patients who received HSCT developed HBV reactivation with genotype B, but all cases had genotype C viral reactivation. A large Japanese cohort study reported that 12.2% and 84.7% of patients with chronic HBV infection had genotype B and genotype C, respectively^28^, indicating that genotype B is a minor genotype in Japan. Interestingly, genotype B constructs with a wild-type core promoter sequence mostly displayed higher replication capacities than the corresponding genotype C constructs due to more efficient transcription of the 3.5-kb RNA^29,30^. Thus, it may be that strong immunosuppressive conditions mediated by HSCT could provide the opportunity for reactivation for genotype C viruses, while genotype B reactivation was achieved in patients in the non-HSCT group due to their ability to replicate at high levels compared with genotype C isolates.

Ultra-deep sequencing revealed that non-synonymous variants were abundant in the reactivated HBV of non-HSCT patients compared to cases with HSCT. Of note, the dN/dS ratios in the S region of the reactivated viruses were significantly higher in the non-HSCT cases than in HSCT or AHB patients. Indeed, HBV S regions with dN/dS ratios greater than one were frequently observed in 11 of 14 (79%) cases in the non-HSCT group, while they were observed in only 3 of 7 (43%) and 1 of 9 (11%) of cases in the HSCT and AHB groups, respectively. These findings suggest that variants with amino acid changes of the S protein were positively selected, especially in patients under immunosuppression without HSCT. Interestingly, amino acid substitutions observed in the reactivated HBV frequently included those putatively associated with impairment of the anti-HBs immune response. For example, 8 of 24 (33%) patients with HBV reactivation after resolved infection had viral isolates with immune escape variants in the S gene. Moreover, 6 of 24 (25%) patients had sL21S and/or sF220C amino acid variants in the MHC class II-restricted T-cell epitope of the small S protein, potentially leading to a complete loss of T-cell reactivity and anti-HBs production^22^. Of note, 10 of 16 (63%) patients in the non-HSCT group had those variants with the small S protein substitutions, including six cases (38%) with immune escape variants, and six (38%) with MHC class II-restricted T-cell epitope variants. These findings suggest that viral isolates that could escape the host’s acquired immunity to impaired reactivity with B cells and/or MHC class II-restricted T cells are frequently involved in the pathogenesis of HBV reactivation after resolved infection, especially in patients receiving immunosuppressive therapies and/or cytotoxic chemotherapies without HSCT. In comparison with reactivated viruses in patients without HSCT, those in patients with HSCT had few amino acid variants and only two of eight (25%) patients had amino acid substitutions associated with impairment of anti-HBV immune responses in the S region. As the previous study revealed that HSCT recipients who originally had latent HBV infection in their livers gradually lost their acquired HBV immunity (reaching 90% loss at 5 years) after HSCT^11^, we suppose that the anti-HBV immune response would differ between patients with HSCT and those without HSCT, leading to the different genetic features of the reactivated HBV.

Several studies demonstrated that specific amino acid substitutions in the S gene of HBV impair virion secretion from cells to the culture supernatant *in vitro*^17,23,27^. In the clinical setting, variants with a phenotype of impaired virion secretion are found in patients chronically infected with HBV. Xiang *et al*. demonstrated that 3 of 230 (1.3%) hepatitis B e antigen-positive chronic hepatitis B patients were infected with sE2G variants, 9 of 230 (3.9%) were infected with sL98V variants, and 3 of 230 (1.3%) were infected with sG145R variants in the small S protein^24^. The virologic and/or pathologic significance of the reduced virion secretion for HBV infection, however, remains unsolved. In the present study, we demonstrated that reactivated HBV frequently contained amino acid substitutions related to impaired virion secretion. Indeed, sE2G, sL77R, sL98V, sT118K, sQ129H, and sG145R, all of which cause reduced virion secretion^17,20,24,25^, were found in the sera from 9 of 24 (38%) patients with HBV reactivation. It is well established that viruses lacking middle S protein production can form a viral structure, but reduce virion secretion^26,27^. In addition, Pollicino *et al*. demonstrated that PreS/S variants caused an imbalance in the synthesis of the S proteins^31^. Interestingly, 4 of 16 (25%) patients in the non-HSCT group developed HBV reactivation by variants with the M1V/I substitution at the PreS2 start codon, leading to ablation of the middle S protein. In contrast, variants in the S and/or PreS2 coding domain associated with reduced virion secretion were detected in only one case in the HSCT group and none in the acute hepatitis patients. Why viral clones with impaired virion secretion were predominantly detected in patients with HBV reactivation, however, remains unknown. One possibility is that the impaired virion secretion from the infected cells reduces the opportunities for circulating viruses to encounter the immune cells in the early phase of HBV reactivation, and thus may afford viruses the chance to acquire additional variants to escape from immune response and/or to replicate more efficiently for viral exacerbation.

An unanswered question is whether all these specific variants in the S gene are acquired after viral reactivation, or whether the latently infected HBV in the liver originally contained these S gene variants. To gain insight into the dynamics of variants during HBV reactivation, we examined the viral genome sequences of S region in the liver of 62 individuals with resolved HBV infection. We found that the prevalence of HBV variants with altered small S protein was significantly lower in the liver of individuals with resolved infection than in the sera of patients with HBV reactivation. These findings suggest that HBV variants with altered small S protein associated with immune escape and/or impaired virion secretion emerged and expanded after viral reactivation. Consistent with this hypothesis, the appearance of HBV variants with S protein substitution was observed in the sera of one representative case with viral reactivation. Taken together, these findings suggest that latently infected HBV clones could acquire several variants in the S region and the viral isolates that acquired the altered small S protein associated with immune escape are selected for and become dominant in the process of HBV reactivation.

In conclusion, ultra-deep sequencing analyses demonstrated differences in the virologic features of the reactivated HBV clones between immunosuppressed patients with and without HSCT. Expansion of viral variants with an accumulation of amino acid substitutions in the S and PreS2 region was frequently observed in cases without HSCT compared to those with strong HSCT-mediated immunosuppression and loss of acquired anti-HBV immunity. These amino acid substitutions in the S and PreS2 region frequently included those associated with immune escape, MHC class II-restricted T-cell epitope alterations, and/or impaired virion secretion. Those variants in the S gene might newly emerge after reactivation and selectively expand under anti-HBs immune pressure in patients with originally resolved HBV infection. From these findings, we supposed that a subset of anti-HBV immune response-related variants could newly emerge during viral exacerbation and play a role in the pathogenesis of HBV reactivation under immunosuppressive conditions, especially in cases receiving immunosuppressive therapy and/or cytotoxic chemotherapy without HSCT.

## Methods

### Patients and Samples

Between April 2007 and December 2016, 24 patients who were HBsAg-negative and anti-HBc-positive prior to the initiation of immunosuppressive therapy or cytotoxic chemotherapy were diagnosed with HBV reactivation at Kyoto University Hospital and affiliated hospitals. All patients were originally HBsAg-negative, but anti-HBc-positive, before HBV reactivation, and lacked any risk factors for external viral transmission. All patients were longitudinally followed up at 0.5–3 month intervals until analysis (November 2017) or death. Twenty-three patients with AHB were randomly selected as controls.

Serum samples were obtained at diagnosis of HBV reactivation based on the appearance of circulating HBV DNA under immunosuppressive conditions. All serum samples were frozen and stored at −80 °C until used in this study. Serologic HBV markers, including HBsAg, anti-HBs, and anti-HBc were measured by chemiluminescent enzyme immunoassay (CLEIA; Fuji Rebio, Tokyo, Japan). The serum HBV DNA titer was analyzed by commercial PCR (COBAS Taqman HBV test; Roche, Branchburg, NJ, USA) with a lower detection limit of 1.3 log IU/ml.

To investigate the viral sequences of the S gene in the liver of individuals with resolved infection, liver tissues were obtained from 90 healthy donors negative for HBsAg and positive for anti-HBc who underwent hepatectomy for LDLT at Kyoto University Hospital. All liver tissues were frozen and stored at −80 °C until used in this study.

The Kyoto University Ethics Committee approved this study, and written informed consent was obtained from all patients. The study was conducted in accordance with the principles of the Declaration of Helsinki.

### Sequencing method

PCR and Sanger sequencing protocols for the viruses from the sera of patients with HBV reactivation and AHB were described previously^12^. Because of the low viral load in the liver of the LDLT donors, nested PCR was performed. To obtain whole S gene sequences, four amplicons were constructed using eight paired primers (Supplementary Table S6).

Ultra-deep sequencing of the HBV genome was conducted using the Ion Proton^TM^ Sequencer (Thermo Fisher Scientific, Waltham, MA, USA). HBV DNA amplicons were quantified using the Invitrogen Qubit^®^ 2.0 Fluorometer (Thermo Fisher Scientific). Libraries were generated using 100 ng of genomic DNA and an Ion Xpress Plus Fragment Library Kit with the Ion Shear Plus Reagents Kit. Amplicons were ligated to adapters from the Ion Xpress^TM^ Barcode Adapters. An Agilent 2200 TapeStation (Agilent Technologies, Santa Clara, CA, USA) was used to visualize the size range, and an Ion Library TaqMan^®^ Quantitation Kit was used to determine the library concentration for preparation of the emulsion PCR. After dilution of each library to 100 pmol/l, 6 µl of the library was used as a template for clonal amplification on Ion Sphere particles during emulsion PCR according to the Ion PI^TM^ Template OT2 200 Kit v3 User Guide. Sequencing was conducted over 520 sequencing cycles with the Ion PI^TM^ Hi-Q^TM^ Sequencing 200 Kit on an Ion PI^TM^ Chip v3 (Thermo Fisher Scientific). The above-mentioned protocol resulted in an average read length of 200–300 nucleotides.

### Sequencing Data Analysis

Using high-performance alignment software, NextGENe^®^ (ver. 2.4.2; SoftGenetics, State College, PA, USA), the sequence reads obtained from the Ion Proton^TM^ Sequencer were aligned with reference sequences for the entire HBV genome (3215 bp) that were determined from direct population Sanger sequencing of each clinical specimen. Reads with ≥90% of bases matching a particular position in the reference sequences were aligned. Two quality filters were used for sequencing reads to remove sequencing or alignment errors. Variations that occurred at largely different frequencies in the forward and reverse directions, and ≥3 times in the 10-bp region on either side were removed. Only sequences that passed the quality filters were analyzed, and each position of the viral genome was assigned a coverage depth, representing the number of times the nucleotide position was sequenced. We defined the cut-off value in the current platform as 1% to exclude mismatch errors and to detect low-abundance mutations according to our previous report^12^.

The subgenotypes of HBV were determined by phylogenetic analysis of the full HBV genome sequences with the software MEGA 7.0 using control strains from the NCBI site (Supplementary Fig. S3).

To determine the nucleotide and amino acid variants, two reference genomes and amino acid sequences of each subgenotype from the NCBI database were used as follows: accession numbers AB246342 and AB900105 for subgenotype B1/Bj, AB246339 and JQ801516 for subgenotype B2/Ba, AB246346 and JQ801515 for subgenotype C1/Cs, and AB246345 and AB697502 for subgenotype C2/Ce. The reference sequences of each subgenotype in our study were mainly selected from a previous study^30^, which clarified the functional differences of each HBV subgenotype by using plasmids carrying HBV genomes extracted from cases with representative sequences from each subgenotype. Nucleotide/amino acid variants were defined when each nucleotide or amino acid of HBV sequences we determined did not exactly coincide with the respective reference alignment of each subgenotype.

The ratios of non-synonymous to synonymous evolutionary changes (dN/dS ratios) were calculated using PAL2NAL (http://www.bork.embl.de/pal2nal/) in each protein^32^. The reference sequences for dN/dS analysis were as follows: accession numbers AB246342 for subgenotype B1/Bj, AB246339 for subgenotype B2/Ba, AB246346 for subgenotype C1/Cs, and AB246345 for subgenotype C2/Ce^30^. To calculate the dN/dS ratios for each protein, cases with fewer than three nucleotide variants were excluded.

Analysis of NLG sites was performed on the S gene sequences of each HBV isolate using the program N-GlycoSite (https://www.hiv.lanl.gov/content/sequence/GLYCOSITE/glycosite.html). NLG targets an amino acid sequence motif that is defined by NXS/T, where X represents any amino acid other than proline^33^.

### Statistical Analysis

All analyses were carried out using IBM SPSS Statistics ver. 24.0 (SPSS Inc., Chicago, IL, USA). The non-parametric Mann–Whitney *U* test was used to compare two continuous variables, and the Kruskal-Wallis test, followed by the Steel-Dwass test for post hoc analysis, was used to compare the three groups. The Fisher exact test was used to compare two categorical variables, and the chi-square test was used to compare three categorical variables. A *P*-value less than 0.05 was considered statistically significant.

## Electronic supplementary material

Supplementary Figure

## Data Availability

Sequence reads with Genome Analyzer were deposited in the DNA Data Bank of Japan Sequence Read Archive (http://trace.ddbj.nig.ac.jp/dra/index_e.shtml) under accession number DRA006491.
