# Supplementary material for: Expansion of viral variants associated with immune escape and impaired virion secretion in patients with HBV reactivation after resolved infection
Source: Sci Rep. 2018 Dec 24;8:18070. doi: 10.1038/s41598-018-36093-w (PMC6305382; doi:10.1038/s41598-018-36093-w)
Supplement: Supplementary file 1 — Supplementary Figure [file 41598_2018_36093_MOESM1_ESM.pdf]

Supplementary Information

Expansion of viral variants associated with immune escape and impaired virion secretion in patients with HBV reactivation after resolved infection

Tadashi Inuzuka[1,2,3], Yoshihide Ueda[1], Soichi Arasawa[1], Haruhiko Takeda[1,3], Tomonori Matsumoto[1,3], Yukio Osaki[4], Shinji Uemoto[5], Hiroshi Seno[1] and Hiroyuki Marusawa[1,4]

[1]Department of Gastroenterology and Hepatology, Graduate School of Medicine, Kyoto University, Kyoto, Japan.

[2]Research Center for Hepatitis and Immunology National Center for Global Health and Medicine, Chiba, Japan

[3]Research Fellow of Japan Society for the Promotion of Science

[4]Department of Gastroenterology and Hepatology, Osaka Red Cross Hospital, Osaka, Japan.

[5]Department of Surgery, Graduate School of Medicine, Kyoto University, Kyoto, Japan

A

HBsAg subregions	Amino acid position	Reference sequences B1	Reference sequences B2	AHB group (genotype B) Patient identification A1	A2	A3
	2	E	E	-	-	-
	3	N	N	-	-	-
	4	I	I	-	-	-
	7	G	G	-	-	-
	8	L	L	-	-	-
MHC Class II	16	Q	Q	-	-	-
	21	L	L	-	-	-
	28	I	I	-	-	-
	29	P	P	-	-	-
	33	D	D	-	-	-
	44	G	E/G	-	-	-
	56	Q	Q	-	-	-
	59	S	S	-	-	-
	73	R	R	-	-	-
Major hydrophilic region	99	D	D	-	-	-
	100	Y	Y	-	-	-
	105	P	P	-	-	-
	109	L	L	-	-	-
	116	T	T	-	-	-
	117	S	S	-	-	-
	118	T	T	-	-	-
	119	G	G	-	-	-
	120	P	P	-	-	-
	121	C	C	-	-	-
	122	K	K	-	-	-
	123	T	T	-	-	-
	126	T	T	-	-	-
	129	Q	Q	-	-	-
	139	C	C	-	-	-
	144	D	D	-	-	-
	145	G	G	-	-	-
	160	K	K	-	-	-
	161	Y	Y	-	-	-
	164	E	E	-	-	-
	165	W	W	-	-	-
	168	V	V	-	-	-
	169	R	R	-	-	-
	174	S	S	-	-	-
	177	V	V	-	-	-
	178	P	P	-	-	-
	181	Q	Q	-	-	-
	185	G	G	-	-	-
	190	V	V	-	-	-
	200	Y	F	-	-	Y
	204	S	S	-	-	-
MHC Class II	213	M	I/M	-	-	-
	214	P	P	-	-	-
	219	F	F	-	-	-
	220	F	F	-	-	-
	224	V	V	-	-	-
	226	I	I	-	-	-

B

HBsAg subregions	Amino acid position	Reference sequences C1	Reference sequences C2	AHB group (genotype C) Patient identification A4	A5	A6	A7	A8	A9	A10	A11	A12	A13	A14	A15	A16	A17	A18	A19	A20	A21	A22	A23
	2	E	E	-	-	-	-	-	-	-	-	-	-	-	-	-	-	-	-	-	-	-	-
	3	S	N/S	-	-	-	-	-	-	-	-	-	-	-	-	-	-	-	-	-	-	-	-
	5	T	T	-	-	-	-	-	-	-	-	-	-	-	-	-	-	-	-	-	-	-	-
	6	S	S	-	-	-	-	-	-	-	-	-	-	-	-	-	-	-	-	-	-	-	-
	8	F	F	-	-	-	-	-	-	-	-	-	-	-	-	-	-	-	-	-	-	-	-
	14	V	V	-	-	-	-	-	-	-	-	-	-	-	A	-	-	-	-	-	-	-	-
MHC Class II	16	Q	Q	-	-	-	-	-	-	-	-	-	-	-	-	-	-	-	-	-	-	-	-
	21	L	L	-	-	-	-	-	-	-	-	-	-	-	-	-	-	-	-	-	-	-	-
	24	K/R	R	-	-	K	-	-	-	-	-	-	-	-	-	-	-	-	-	-	-	-	-
	30	Q	Q	-	-	-	-	-	-	-	-	-	-	-	K	-	-	-	-	-	-	-	-
	31	S	S	-	-	-	-	-	-	-	-	-	-	-	-	-	-	-	-	-	-	-	-
	33	D	D	-	-	-	-	-	-	-	-	-	-	-	-	-	-	-	-	-	-	-	-
	34	S	S	-	-	-	-	-	-	-	-	-	-	-	-	-	-	-	-	-	-	-	-
	40	N	N	-	-	-	-	-	-	-	-	-	-	-	-	-	-	-	-	-	-	-	-
	41	F	F	-	-	-	-	-	-	-	-	-	-	-	-	-	-	-	-	-	-	-	-
	45	A	A	-	-	-	-	-	-	-	-	-	-	-	T	-	-	-	-	-	-	-	-
	47	T	T	-	-	-	-	-	-	-	-	-	-	-	A	-	-	-	-	-	-	-	-
	53	L/S	S	-	-	-	-	-	-	-	-	-	-	-	-	-	-	L	-	-	-	-	-
	57	T	T	-	-	-	-	-	-	-	-	-	-	-	-	-	-	-	-	-	-	-	-
	67	P	P	-	-	-	-	-	-	-	-	-	-	-	-	-	-	-	-	-	-	-	-
	74	W	W	-	-	L	-	-	-	-	-	-	-	-	-	-	-	-	-	-	-	-	-
	77	L	L	-	-	-	-	-	-	-	-	-	-	-	-	-	-	-	-	-	-	-	-
	80	F	F	-	-	-	-	-	-	-	-	-	-	-	-	-	-	-	-	-	-	-	S
	94	L	L	-	-	-	-	-	-	-	-	-	-	-	-	-	-	-	-	-	-	-	-
	96	V	V	-	-	-	-	-	-	-	-	-	-	-	-	-	-	-	-	-	-	-	-
	97	L	L	-	-	-	-	-	-	-	-	-	-	-	-	-	-	-	-	-	-	-	-
	98	L	L	-	-	-	-	-	-	-	-	-	-	-	-	-	-	-	-	-	-	-	-
Major hydrophilic region	99	D	D	-	-	-	-	-	-	-	-	-	-	-	-	-	-	-	-	-	-	-	-
	100	Y	Y	-	-	-	-	-	-	-	-	-	-	-	C	-	-	-	-	-	-	-	-
	101	Q	Q	-	-	-	-	-	-	-	-	-	-	-	-	-	-	-	-	-	-	-	-
	109	L	L	-	-	-	-	-	-	-	-	-	-	-	-	-	-	-	-	-	-	-	-
	116	T	T	-	-	-	-	-	-	-	-	-	-	-	-	-	-	-	-	-	-	-	-
	117	S	S	-	-	-	-	-	G	-	-	-	-	-	-	-	-	-	-	-	-	-	-
	118	T	T	-	-	-	-	-	-	-	-	-	-	-	-	-	-	-	-	-	-	-	-
	123	T	T	-	-	-	-	-	-	-	-	-	-	-	-	-	-	-	-	-	-	-	-
	126	I/T	I	-	-	-	-	-	-	-	-	T	-	-	T	-	-	-	-	-	-	-	-
	127	P	P	-	-	-	-	-	-	-	-	-	-	-	-	-	-	-	-	-	-	-	-
	129	Q	Q	-	-	-	-	-	-	-	-	-	-	-	-	-	-	-	-	-	-	-	-
	130	G	G	-	-	-	-	-	-	-	-	-	-	-	-	-	-	-	-	-	-	-	-
	143	S	S	-	-	-	-	-	-	-	-	-	-	-	-	-	-	-	-	-	-	-	-
	145	G	G	-	-	-	-	-	-	-	-	-	-	-	-	-	-	-	-	-	-	-	-
	157	A	A	-	-	-	-	-	-	-	-	-	-	-	-	-	-	-	-	-	-	-	-
	159	A	A	-	-	-	-	-	-	-	-	-	-	-	-	-	-	-	-	-	-	-	-
	161	F	F	-	-	-	-	-	-	V	-	-	-	-	-	-	-	-	-	-	-	-	-
	164	E	E	-	-	-	-	-	-	-	-	-	-	-	-	-	-	-	-	-	-	-	-
	167	S	S	-	-	-	-	-	-	-	-	-	-	-	-	-	-	-	-	-	-	-	-
	168	V	V	-	-	-	-	-	-	-	-	-	-	-	-	-	-	-	-	-	-	-	-
	169	R	R	-	-	-	-	-	-	-	-	-	-	-	-	-	-	-	-	-	-	-	-
	172	W	W	-	-	-	-	-	-	-	-	-	-	-	-	-	-	-	-	-	-	-	-
	174	S	S	-	-	-	-	-	-	-	-	-	-	-	-	-	-	-	-	-	-	-	-
	175	L	L	-	-	-	-	-	-	-	-	-	-	-	-	-	-	-	-	-	-	-	-
	177	V	V	-	-	-	-	-	-	-	-	-	-	-	-	-	A	-	A	-	-	-	-
	184	V	V	-	-	-	-	A	-	A	A	A	-	-	-	-	-	-	-	-	A	-	-
	190	V	V	-	-	-	-	-	-	-	-	-	-	-	-	-	-	-	-	-	-	-	-
	196	W	W	-	-	-	-	-	-	-	-	-	-	-	-	-	-	-	-	-	-	-	-
	200	Y	Y	-	-	-	-	-	-	-	-	-	-	-	-	-	-	-	-	-	-	-	-
	203	P	P	-	-	-	-	-	-	-	-	-	-	-	-	-	-	-	-	-	-	-	-
MHC Class II	213	L	L	-	-	-	-	-	-	-	-	-	-	-	-	-	-	-	-	-	-	-	-
	220	F	F	-	-	-	-	-	-	-	-	-	-	-	-	-	-	-	-	-	-	-	-
	223	W	W	-	-	-	-	-	-	-	-	-	-	-	-	-	-	-	-	-	-	-	-
	225	Y	Y	-	-	-	-	-	-	-	-	-	-	-	-	-	-	-	-	-	-	-	-
	226	I	I	-	-	-	-	-	-	-	-	-	-	-	-	-	-	-	-	-	-	-	-

Supplementary Fig. S1. Amino acid variants of the small S protein in patients with acute hepatitis B.

Amino acid variants were determined by the same method as in Fig. 2. The amino acids harbored by each reference sequence are shown on the left side. Only positions with amino acid substitutions as compared with HBV reference sequences are shown, and the dash indicates the absence of an amino acid substitution. A1-23 are the cases with AHB. (A) Patients with genotype B HBV. (B) Patients with genotype C HBV.

Abbreviation: AHB, acute hepatitis B

A

PreS domain	Amino acid position	Reference sequences: B1	Reference sequences: B2	AHB group (genotype B), Patient identification: A1	A2	A3
PreS1	1	M	M	-	-	-
	7	K	K	-	-	-
	44	D	D	-	-	-
	47	P	P	-	-	L
	51	N	N	-	-	-
	54	D	D	-	E	-
	55	A	A	-	-	-
	62	A	A	-	-	-
	79	P	P	-	-	-
	84	I	L	-	-	I
	91	A	A	-	T	-
	97	T	T	-	-	-
	98	N	N	-	-	-
	104	Q	Q	-	-	-
	108	L	L	-	-	-
	109	S	S	-	-	-
	119	A	A	-	-	-
PreS2	1	M	M	-	-	-
	2	Q	Q	-	-	-
	7	T	T	-	-	-
	9	H	H	-	-	-
	10	Q	Q	-	-	-
	13	Q	Q	-	-	-
	15	P	P	-	-	-
	41	A	A	-	-	-
	55	N	N	-	-	-

B

PreS domain	Amino acid position	Reference sequences: C1	Reference sequences: C2	AHB group (genotype C), Patient identification: A4	A5	A6	A7	A8	A9	A10	A11	A12	A13	A14	A15	A16	A17	A18	A19	A20	A21	A22	A23
PreS1	1	M	M	-	-	-	-	-	-	-	-	-	-	-	-	-	-	-	-	-	-	-	-
	4	W	W	-	-	-	-	-	-	-	-	-	-	-	-	-	Y	-	-	-	-	-	-
	5	S	S	-	-	-	-	-	-	-	-	-	-	-	-	-	-	-	-	-	-	-	-
	7	K	K	-	T	-	-	-	-	-	-	-	-	-	-	-	-	-	-	-	-	-	-
	10	Q	Q	-	-	-	-	-	-	-	-	-	-	-	K	-	K	-	-	-	-	-	-
	25	F	F	-	-	-	-	-	-	-	-	-	-	-	-	-	L	-	-	-	-	-	-
	35	G	G	-	-	-	-	-	-	-	-	-	-	-	-	-	-	-	-	-	-	-	-
	39	N	N	-	-	-	-	-	-	-	-	-	-	-	-	-	-	-	-	-	-	-	-
	51	Q	H	-	-	-	-	-	-	-	-	-	-	-	-	-	P	-	-	-	Q	-	-
	54	A	A	-	-	-	-	-	-	-	-	-	-	-	-	-	E	-	-	-	-	-	-
	56	N	N	-	-	-	T	-	-	-	-	-	-	-	-	-	W	-	-	-	-	-	T
	60	V	A	-	-	-	-	-	-	-	-	-	-	-	-	-	A	-	-	-	V	-	-
	65	P	P	-	-	-	S	-	-	-	-	-	-	-	-	S	-	-	-	-	-	-	-
	67	F	F	-	-	-	-	-	-	-	-	-	-	-	V	-	-	-	-	-	-	-	-
	70	P	P	-	-	-	-	-	-	-	-	-	-	-	-	-	-	-	-	-	-	-	-
	73	N/S	G	-	-	-	-	-	-	-	-	-	-	-	-	-	G	-	-	-	-	-	-
	82	Q	Q	-	-	-	-	-	-	-	-	-	-	-	-	-	-	-	-	-	-	-	-
	83	G	G	-	-	-	-	-	-	-	-	-	-	-	-	-	-	-	-	-	-	-	-
	84	I	I/V	-	-	-	-	-	-	T	-	-	-	-	-	-	-	-	-	-	-	-	T
	85	L	L	-	-	-	-	-	-	-	-	-	-	-	-	-	-	-	-	-	-	-	-
	87	T	T	-	-	-	-	-	-	-	-	-	-	-	S	-	-	-	-	-	-	-	-
	88	V	V	-	-	-	-	-	-	-	-	-	-	-	-	-	-	-	-	-	-	-	-
	89	P	P	-	-	-	-	-	-	-	-	-	-	-	-	-	-	-	-	-	-	-	-
	90	A	V	A	A	A	A	A	-	A	A	A	-	-	A	A	-	-	A	-	A	A	-
	96	S	S	-	-	-	-	-	-	-	-	-	-	-	-	-	-	-	-	-	-	-	-
	104	Q	Q	-	-	-	-	-	-	-	-	-	-	-	-	-	-	-	-	-	-	-	-
	114	D	D	-	-	-	-	-	-	-	-	-	-	-	-	-	-	-	-	-	-	-	-
	119	A	A	-	-	-	-	-	-	-	-	-	-	-	-	-	-	-	-	-	-	-	-
PreS2	1	M	M	-	-	-	-	-	-	-	-	-	-	-	-	-	-	-	-	-	-	-	-
	2	Q	Q	-	-	-	-	-	-	-	-	-	-	-	-	-	-	-	-	-	-	-	-
	13	L	L	-	-	-	-	-	-	-	-	-	-	-	-	-	-	-	-	-	-	-	-
	14	D	D	-	-	-	-	-	-	-	-	-	-	-	-	-	-	-	-	-	-	-	-
	19	G	G	-	-	-	-	-	-	-	-	-	-	-	-	-	-	-	-	-	-	-	-
	21	F/L	Y	-	-	-	-	-	-	-	-	-	-	-	-	-	-	-	-	-	-	-	-
	32	V	V	-	-	-	-	A	-	-	-	I	-	-	-	-	A	-	-	-	-	-	-
	37	T	T	-	-	-	-	-	-	-	I	-	-	-	-	-	-	-	-	-	-	-	-
	39	A	A	-	-	-	-	-	-	-	-	-	-	-	-	-	-	-	-	-	-	-	-
	45	I	I	-	-	-	-	T	-	-	-	-	-	-	-	-	-	-	-	-	-	T	-
	48	R	R	-	-	-	-	-	-	-	-	-	-	-	-	K	-	-	-	-	-	-	-
	49	T	T	I	-	-	-	I	-	-	-	-	-	-	-	-	-	-	-	-	-	-	-
	52	P	P	-	-	-	-	-	-	-	-	-	-	-	-	-	-	-	-	-	-	-	-
	55	N	N	-	-	-	-	-	-	-	-	-	-	-	-	-	-	-	-	-	-	-	-

Supplementary Fig. S2. Amino acid variants of the PreS1/PreS2 coding domain in patients with acute hepatitis B.

Amino acid variants were determined by the same method as in Fig. 2. Amino acids harbored by each reference sequence are shown on the left side. Only positions with amino acid substitutions as compared with HBV reference sequences are shown, and the dash indicates the absence of an amino acid substitution. A1-23 are the cases with AHB. (A) Patients with genotype B HBV. (B) Patients with genotype C HBV.
Abbreviation: AHB, acute hepatitis B, PreS, presurface

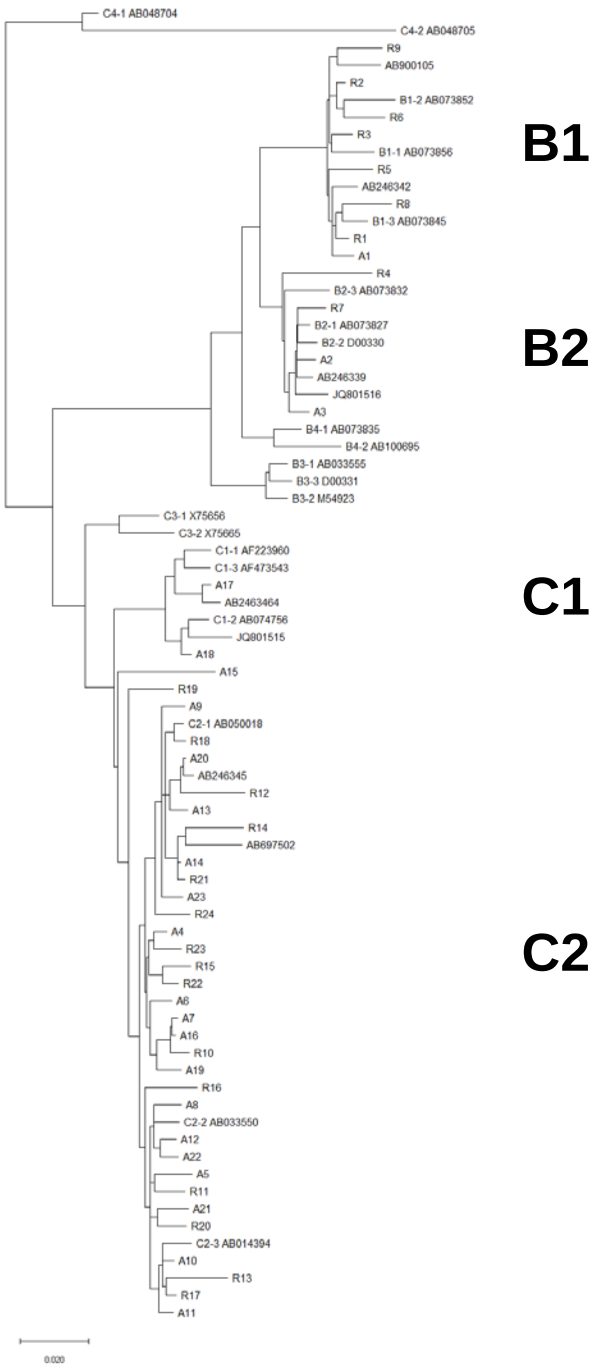

Supplementary Fig. S3. The Phylogenetic analysis by Maximum Likelihood method.

The evolutionary history was inferred by using the Maximum Likelihood method based on the Tamura-Nei model. The tree is drawn to scale, with branch lengths measured in the number of substitutions per site. Evolutionary analyses were conducted in MEGA 7.0.

Supplementary Table S1

The average number of aligned reads on the whole HBV genome and mean coverage at a nucleotide position of patients with HBV reactivation and acute hepatitis B determined by ultra-deep sequencing

	Average aligned reads	Average coverage
non-HSCT (n=16)	1,596,283	53,636
HSCT (n=8)	1,552,168	62,132
AHB (n=23)	1,413,000	48,025

Supplementary Table S2

The number of nucleotide variants which accounted for more than 5% and 20% of the heterogeneity in the whole HBV nucleotides

Case	Variant frequency >5% (/3215 bases)	Variant frequency >20% (/3215 bases)
**R1**	7	1
**R2**	6	0
**R3**	13	5
**R4**	79	42
**R5**	13	0
**R6**	27	14
**R7**	8	3
**R8**	10	8
**R9**	5	5
**R10**	12	2
**R11**	2	2
**R12**	73	32
**R13**	18	6
**R14**	1	1
**R15**	53	22
**R16**	5	3
**R17**	10	2
**R18**	7	3
**R19**	0	0
**R20**	4	0
**R21**	0	0
**R22**	3	0
**R23**	0	0
**R24**	44	44
**median**	7.5	2.5

Supplementary Table S3

The average number of aligned reads on the HBV surface gene and mean coverage at a nucleotide position of individuals with latent liver infection determined by ultra-deep sequencing

	Average aligned reads	Average coverage
HBV latently infected in the liver (n=60)	305,775	36,047

Supplementary Table S4

Baseline clinical characteristics of 60 individuals with resolved HBV infection

**Patients' Characteristics**	**Individuals with resolved HBV infection (n=60)**
**Gender** (M/F)	24/36
**Median age** (years)	46 (20-61)
**HBV status**, N(%)	
Isolated anti-HBc positive	11 (18%)
Anti-HBc positive/anti-HBs positive	49 (82%)
**HBV subgenotype**, N(%)	
B1/Bj	3 (5%)
B2/Ba	5 (8%)
C1/Cs	0 (0%)
C2/Ce	52 (87%)

Supplementary Table S5: The frequencies of specific amino acid substitutions in the S gene region among viruses latently infected in the liver of individuals with resolved HBV infection

Patient Identification	E2G	L21S	L77R	L98V	T116N	T118K	T123N	Q129H	G130N	G145A/R	F220C
#1	-*	-	-	-	-	-	-	-	-	-	-
#2	-	-	-	-	-	-	-	-	-	-	-
#3	-	-	-	-	-	-	-	-	-	-	-
#4	-	-	-	-	-	-	-	-	-	-	-
#5	-	-	-	-	-	-	-	-	-	-	-
#6	-	99.9%	-	-	-	-	-	-	-	-	-
#7	-	-	-	-	-	-	-	-	-	-	-
#8	99.6%	-	-	-	-	-	-	-	-	-	-
#9	-	-	-	-	-	-	-	-	-	-	-
#10	-	-	-	-	-	-	-	-	-	-	-
#11	-	-	-	-	-	-	-	-	-	1.9%	-
#12	-	-	-	-	-	-	-	-	-	-	-
#13	-	-	-	-	-	-	-	-	-	-	-
#14	-	-	-	-	-	-	-	-	-	-	-
#15	-	-	-	-	-	-	-	-	-	-	-
#16	-	-	-	-	-	-	-	-	-	-	-
#17	-	-	-	-	-	-	-	-	-	-	-
#18	-	-	-	-	-	-	-	-	-	-	-
#19	-	-	-	-	-	-	-	-	-	-	-
#20	-	-	-	-	-	-	-	-	-	-	-

Patient Identification	E2G	L21S	L77R	L98V	T116N	T118K	T123N	Q129H	G130N	G145A/R	F220C
#21	-	-	-	-	-	-	-	-	-	-	-
#22	-	-	-	-	-	-	-	-	-	-	-
#23	-	-	-	-	-	-	-	-	-	-	-
#24	-	-	-	-	-	-	-	-	-	-	-
#25	-	-	-	-	-	-	-	-	-	-	-
#26	-	-	-	-	-	-	-	-	-	-	-
#27	-	-	-	-	-	-	-	-	-	-	-
#28	-	-	-	-	-	-	-	-	-	-	-
#29	-	-	-	-	-	-	-	-	-	-	-
#30	-	-	-	-	-	-	-	-	-	-	-
#31	-	-	-	-	-	-	-	-	-	-	-
#32	-	-	-	-	-	-	-	-	-	-	-
#33	-	-	-	-	-	-	-	-	-	-	-
#34	-	-	-	-	-	-	-	-	-	-	-
#35	-	-	-	-	-	-	-	-	-	-	-
#36	-	-	-	-	-	-	-	-	-	-	-
#37	-	-	-	-	-	-	-	-	-	-	-
#38	-	-	-	-	-	-	-	-	-	-	-
#39	-	-	-	-	-	-	-	-	-	-	-
#40	-	-	-	-	-	-	-	-	-	-	-

Patient Identification	E2G	L21S	L77R	L98V	T116N	T118K	T123N	Q129H	G130N	G145A/R	F220C
#41	-	-	-	-	-	-	-	-	-	-	-
#42	-	-	-	67.3%	-	-	-	-	-	-	-
#43	-	-	-	-	-	-	-	-	-	-	-
#44	-	-	-	-	-	-	-	-	-	-	-
#45	-	-	-	-	-	-	-	-	-	-	-
#46	-	-	-	-	-	-	-	-	-	-	-
#47	-	-	-	-	-	-	-	-	-	-	-
#48	-	-	-	-	-	-	-	-	-	-	-
#49	-	-	-	-	-	-	-	-	-	-	-
#50	-	-	-	-	-	-	-	-	-	-	-
#51	-	-	-	-	-	-	-	-	-	-	-
#52	-	-	-	-	-	-	-	-	-	-	-
#53	-	-	-	-	-	-	-	-	-	-	-
#54	-	-	-	-	-	-	-	-	-	-	-
#55	-	-	-	-	-	-	-	-	-	-	-
#56	-	-	-	-	-	-	-	-	-	-	-
#57	-	-	-	-	-	-	-	-	-	-	-
#58	-	5.6%	-	-	-	-	-	-	-	-	-
#59	-	-	-	-	-	-	-	-	-	-	-
#60	-	-	-	-	-	-	-	-	-	-	-

* A dash indicates the frequency less than 1%.

Supplementary Table S6

The oligonucleotide primers for amplifying the nucleotide sequences

Primer	Sequence	Usage
PCRS1_out_Fw	cctgctggtggctccagttc	Nested PCR for amplicon 1
PCRS1_out_Rv	gagaagtccaccacgagtc	Nested PCR for amplicon 1
PCRS1_in_Fw	tcgtcaatcttctcgaggac	Nested PCR for amplicon 1
PCRS1_in_Rv	gtctagactctgtggtattgtgag	Nested PCR for amplicon 1
PCRS2_out_Fw	atcaggattcctaggaccc	Nested PCR for amplicon 2
PCRS2_out_Rv	aggacaaacgggcaacatac	Nested PCR for amplicon 2
PCRS2_in_Fw	gcggggtttttcttgttgac	Nested PCR for amplicon 2
PCRS2_in_Rv	gaaccaacaagaagatgaggc	Nested PCR for amplicon 2
PCRS3_out_Fw	tatcgctggatgtgtctgc	Nested PCR for amplicon 3
PCRS3_out_Rv	ccactgaacaaatggcactag	Nested PCR for amplicon 3
PCRS3_in_Fw	gcctcatcttcttgttggttc	Nested PCR for amplicon 3
PCRS3_in_Rv	gaggcccactcccatagg	Nested PCR for amplicon 3
PCRS4_out_Fw	catcatcctgggctttcgc	Nested PCR for amplicon 4
PCRS4_out_Rv	gttcctgtggtaaagtaccc	Nested PCR for amplicon 4
PCRS4_in_Fw	cgcaagattcctatgggagtg	Nested PCR for amplicon 4
PCRS4_in_Rv	cgtttggttttattagggttc	Nested PCR for amplicon 4
